# PCOS Influences the Expression of AMHRII in the Endometrium of AEH During the Reproductive Age

**DOI:** 10.3390/diagnostics14242872

**Published:** 2024-12-20

**Authors:** Yingsha Yao, Shulan Zhu, Xiaoming Zhu

**Affiliations:** 1 Women’s Hospital, School of Medicine, Zhejiang University, Hangzhou 310006, China; yaoyingsha@zju.edu.cn (Y.Y.); ilovelittlepig@163.com (S.Z.); 2 Hangzhou Linping District Maternal & Child Health Care Hospital, Hangzhou 311199, China; 3 Women’s Reproductive Health Key Laboratory of Zhejiang Province, Hangzhou 310006, China

**Keywords:** polycystic ovary syndrome, anti-Müllerian hormone receptor type II, atypical endometrial hyperplasia, reproductive age

## Abstract

Background: Endometrial proliferative lesions (EPLs) encompass endometrial hyperplasia (EH) and endometrial carcinoma (EC). Atypical endometrial hyperplasia (AEH) is associated with an elevated risk of progression to EC. Patients with polycystic ovarian syndrome (PCOS) exhibit higher serum levels of anti-Müllerian hormone (AMH) and a correspondingly increased incidence of EPLs. AMH has the capacity to inhibit the cell proliferation of EPLs derived from Müllerian duct tissue through the AMH-AMH receptor (AMHR) signaling pathway. Methods: Pairs of samples matched by preference scores were randomly selected. Immunohistochemistry was employed to assess the expression levels of AMHR type II (AMHR2) in endometrial tissue. A comparative analysis was performed between tissues from individuals with PCOS and those without, as well as between a normal endometrium and endometrial tissue from individuals with EPLs. This study aimed to elucidate differences in AMHR2 expression among these tissue types. By focusing on AMHR2 expression, the impact of the PCOS-related background on the endometrial AMH-AMHR cascade signaling pathway was initially investigated. Results: The AMHR2 protein was expressed in the endometrium of both the PCOS group and the non-PCOS group during the reproductive age (20–39 years). The expression of the AMHR2 protein in the AEH endometrium of PCOS patients did not differ significantly from that in the normal endometrium of PCOS patients; however, it was significantly higher than in the AEH endometrium of non-PCOS patients (*p* = 0.011). Conversely, the expression of the AMHR2 protein in the AEH endometrium of non-PCOS patients was significantly lower than that in the normal endometrium of non-PCOS patients (*p* = 0.021). Notably, there was no significant difference in AMHR2 protein expression in a normal endometrium between PCOS and non-PCOS patients. Conclusions: The involvement of the endometrial AMH-AMHR cascade signaling pathway and its biological effects in the pathogenesis of AEH are evident. The pathophysiological conditions associated with PCOS, such as elevated serum AMH levels and other pathological states, may directly or indirectly influence the AMH-AMHR cascade signaling pathway in the endometrium. This influence could contribute to the progression of AEH.

## 1. Background

Endometrial proliferative lesions (EPLs) refer to widespread growth abnormalities in the endometrium. EPLs are characterized by endometrial glandular hyperplasia and/or an increased glandular-to-stromal ratio and include both endometrial hyperplasia (EH) and endometrial carcinoma (EC). The development of EPLs is typically attributed to the absence of periodic progesterone effects resulting from the chronic estrogen stimulation to which the endometrium is exposed [1]. According to the 2020 World Health Organization (WHO) Classification Criteria for Female Reproductive Organs Oncology, EH is pathologically classified into atypical endometrial hyperplasia (AEH) and endometrial hyperplasia without atypia (non-AEH) [2]. Both AEH and non-AEH are recognized as precancerous lesions of EC, rather than as early stages of EC itself, with AEH posing a greater risk of progression to EC [3,4].

Anti-Müllerian hormone (AMH), also known as the Müllerian inhibitor, is a 140 kDa isodiosaccharide protein produced by secondary ovarian follicles, presinus follicles, and sinus follicles measuring 8 to 10 mm in diameter or less [5,6]. AMH plays a crucial role in the degeneration of Müllerian ducts during early embryonic development [5,6]. Additionally, as aging occurs and ovarian maturation progresses, AMH is involved in follicle growth and the regulation of the follicular stimulating hormone (FSH) threshold, inhibiting the FSH-induced growth of presinus follicles. Furthermore, AMH can inhibit cell proliferation stimulated by the epidermal growth factor and is significant in regulating cell proliferation, differentiation, programmed cell death, and tissue regeneration [5,6,7,8].

AMH interacts with two similar yet distinct receptors, AMH receptor type I (AMHR1) and AMH receptor type II (AMHR2), to exert its biological activity. Among these, AMHR1 serves as a signaling receptor with various isomers, including Alk2, Alk3, and Alk6 [9], which interact with AMHR2. AMHR2 is responsible for ensuring the specificity of ligand binding; its primary function is to recognize AMH and facilitate signaling [10,11]. The biological effects of the AMH-AMHR signaling cascade can only be fully realized when the signaling pathway is intact and all components function properly.

The expression level of AMHR2 in endometrial tissues has been reported to correlate with the atypia of epithelial cells. Specifically, AMHR2 expression is low in a normal endometrium, while it is significantly elevated in EH and EC [8,12,13]. Notably, previous studies have not explored the relationship between AMHR2 expression levels in endometrial tissue, serum AMH levels, and EPLs in women of reproductive age [8,12,13,14]. Numerous epidemiological studies indicate that women with polycystic ovarian syndrome (PCOS) exhibit higher serum AMH levels [15,16] and a relatively increased incidence of EPLs [17,18,19]. This study focused on AEH as a representative type of EPL, examining the differences in endometrial AMHR2 expression levels between AEH patients with a PCOS background and those without. The objective of this study was to evaluate these differences to gain insights into the underlying mechanisms that drive changes in endometrial AMHR2 expression levels during the progression of AEH.

## 2. Methods

This study utilized a retrospective diagnostic study design. The study was approved by the Ethical Review Committee of Women’s Hospital, School of Medicine, Zhejiang University (Hangzhou, China) for clinical research (Approval No. IRB-20210312-R). The subjects included in this study were women of reproductive age who visited the study support unit and underwent endometrial histopathology between August 2017 and October 2022.

Previous studies conducted by our team have demonstrated that EPL patients of reproductive age (20–39 years) exhibit lower serum AMH levels [20]. In contrast, the serum AMH levels in clinically diagnosed PCOS women are significantly higher than those in non-PCOS women, with the serum AMH levels in clinically diagnosed PCOS women being notably elevated compared to their non-PCOS counterparts [15,16]. In this study, we designed a body environment stratified paired case–control analysis to assess AMHR2 expression levels in the endometrial tissues of women of reproductive age, distinguishing between those with PCOS and non-PCOS. We considered AEH as a representative lesion type of EPL and investigated the correlation between the expression level of AMHR2 in the endometrial tissue of AEH and the occurrence and progression of AEH, taking into account the significant differences in serum AMH levels between PCOS and non-PCOS women.

### 2.1. Inclusion and Exclusion Criteria for Study Subjects

#### 2.1.1. Inclusion Criteria for PCOS Subjects

(1) The diagnostic criteria for PCOS were based on the Rotterdam criteria, which were recommended by the PCOS Expert Group of the European Society of Human Reproduction and Embryology and the American Society for Reproductive Medicine in 2003 [21]. The PCOS phenotype is characterized by three primary features: hyperandrogenism (HA), ovulatory dysfunction (OD), and polycystic ovarian morphology (PCOM), which are categorized into four distinct groups [22]. (2) The age range of the subjects studied was 20 to 39 years. (3) The pathological examination adhered to the diagnostic criteria set forth by the WHO for Female Reproductive System Tumors (2020 edition) [2]. (4) Serum AMH levels were measured at the research unit within three months prior to endometrial sampling, yielding effective detection results.

#### 2.1.2. Exclusion Criteria for PCOS Subjects

These comprised (1) incomplete medical records; (2) a history of ovarian disease and/or ovarian surgery, including premature ovarian insufficiency (POI) and its high-risk subjects; (3) a history of endocrine tumors; (4) the use of steroid hormones, gonadotropins, gonadotropin-releasing hormone, ovulation inducers, or estrogen antagonists during the first three months of specimen collection; (5) the presence of definite abnormalities in the chromosome number or structure or genetic diseases related to ovarian function; (6) complications with other malignant tumors or prior treatment with chemotherapy or abdominal radiotherapy; (7) complications with diseases affecting major organs, such as the cardiovascular system, lungs, liver, kidneys, etc.; and (8) metastatic endometrial tumors.

The diagnostic criteria for POI adhered to the standards recommended by the European Society of Human Embryology and Reproduction [23]. High-risk subjects for POI were defined according to the recommended criteria for sub-clinical POI [24], a decreased ovarian reserve [25], and relevant criteria for a low ovarian response [26,27].

#### 2.1.3. Inclusion and Exclusion Criteria for Non-PCOS Subjects

The diagnostic criteria for PCOS were revised to serve as exclusion criteria, while the remaining inclusion and exclusion criteria remained consistent with those applied to PCOS subjects.

### 2.2. The Paired Sampling Method of the Subjects

The subjects with PCOS and non-PCOS, selected based on the established inclusion and exclusion criteria, were matched in a 1:1 ratio between the AEH subjects and their corresponding control subjects using the propensity score matching method [28]. The matching factors for PCOS subjects included five major confounders associated with serum AMH levels, as outlined in Supplementary Table S1: pregnancy history, body mass index, a PCOS phenotype, hypertension, and diabetes. In contrast, the matching factors for non-PCOS subjects consisted of seven major confounders related to serum AMH levels, as detailed in Supplementary Table S2: age, body mass index (BMI), the carcinoma embryonic antigen (CEA), menstrual regularity, dysmenorrhea, hypertension, and diabetes.

Among the paired subjects, 9 pairs of PCOS and 9 pairs of non-PCOS subjects (AEH lesions versus a normal endometrium) were identified through simple random sampling utilizing the sampling tool from the Excel analysis tool library. Subsequently, endometrial specimens from these 36 subjects were collected, and the expression levels of AMHR2 were compared.

### 2.3. Immunohistochemical Detection of Endometrial AMHR2 Protein

All samples were obtained through a hysteroscopic endometrial biopsy. AMHR2 was extracted from paraffin sections and assessed using immunohistochemistry (IHC) [29,30]. The AMHR2 antibody was obtained from Abcam, Cambridge, MA, USA. The expression level of the AMHR2 protein in the endometrial stroma and glands was determined by randomly selecting three high-power fields in each section. Endometrial stromal cells and adenocarcinoma cells displaying brown-yellow particles in their membranes and cytoplasm were classified as positive cells, while those without staining were considered negative. The cell morphology and staining were examined under a microscope. Phosphate-buffered saline served as the negative control, while a granulosa cell tumor of the ovary was utilized as the positive control.

The semi-quantitative evaluation method for IHC staining in this study was conducted according to the procedures outlined by Hao Dongdong et al. [29,30]. A positive signal was identified by the presence of brown-yellow particles in the cell membrane and cytoplasm. For each section, three high-power visual fields were randomly selected, and Image-Pro Plus software 6.0 (Media Cybernetics Corporation, Rockville, MD, USA) was employed to measure the integral optical density per area (IOD/Area) of the IHC positive expression. Each case was repeated three times, and the mean value was calculated. The expression level of the AMHR2 protein was reported as the corresponding average gray value with the standard deviation (X ± SD), accompanied by relevant statistical analysis.

### 2.4. Statistical and Analytical Methods

Data analysis was conducted using SPSS version 26.0 statistical software. When the data met the assumptions of normality, the paired sample *t* test was employed. Conversely, when the data did not conform to a normal distribution, the paired sample Wilcoxon rank sum test was utilized. A *p* value of less than 0.05 was deemed statistically significant for two-tailed tests.

## 3. Results

### 3.1. The Basic Information of the Research Object

#### 3.1.1. Characteristics of PCOS Subjects

**Figure 1:**
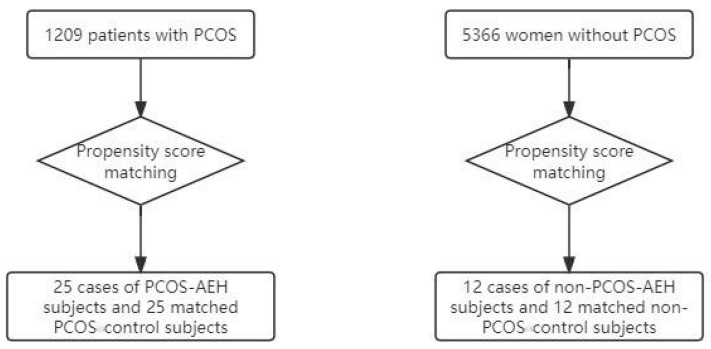
Flow-chart regarding the propensity score analysis.

The study included a total of 1209 patients with PCOS, comprising 55 cases of non-AEH, 29 cases of AEH, and 6 cases of EC. Nine cases of PCOS-AEH subjects and nine matched PCOS control subjects were selected. For a detailed explanation of the propensity score matching procedure, please refer to Figure 1.

The five major confounders associated with serum AMH levels—namely, pregnancy history, BMI, a PCOS phenotype, hypertension, and diabetes—were analyzed in both groups, with the comparative results presented in Table 1.

As indicated in Table 1, the subjects in both groups were comparable concerning these five major confounders related to serum AMH levels, and the differences between the groups were not statistically significant.

#### 3.1.2. Characteristic of Non-PCOS Subjects

The study included a total of 5366 women without PCOS, comprising 81 cases of non-AEH, 30 cases of AEH and 6 cases of EC. Nine non-PCOS-AEH subjects and nine matched non-PCOS-control subjects were selected.

The seven major confounders associated with serum AMH levels—age, BMI, CEA, menstrual regularity, dysmenorrhea history, hypertension, and diabetes—were examined in both groups, with the comparative results presented in Table 2.

As indicated in Table 2, the subjects in both groups were closely matched across these seven major confounders, and the differences between the groups were not statistically significant.

### 3.2. Changes in AMHR2 Expression in AEH Endometrial Specimens of PCOS

**Figure 2:**
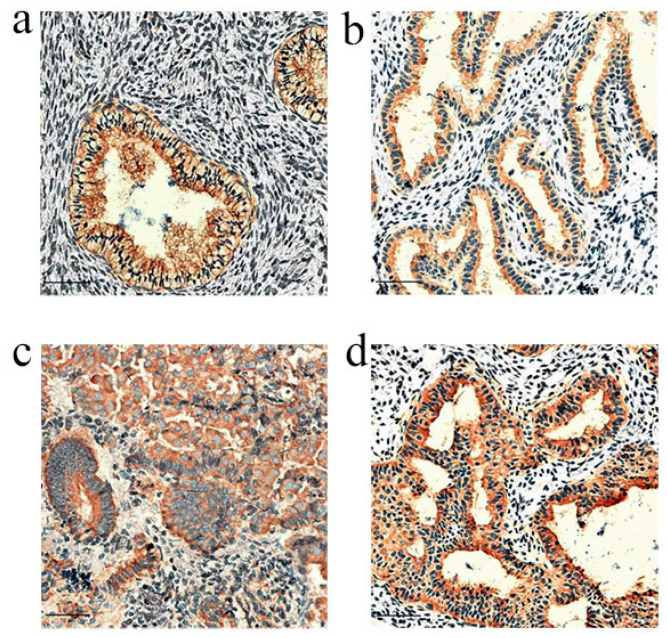
Immunohistochemical staining of AMHR2 in the endometrium of subjects with and without PCOS (scale bar: 50 μm). (**a**) PCOS control group, (**b**) non-PCOS control group, (**c**) PCOS with AEH, (**d**) non-PCOS with AEH.

**Figure 3:**
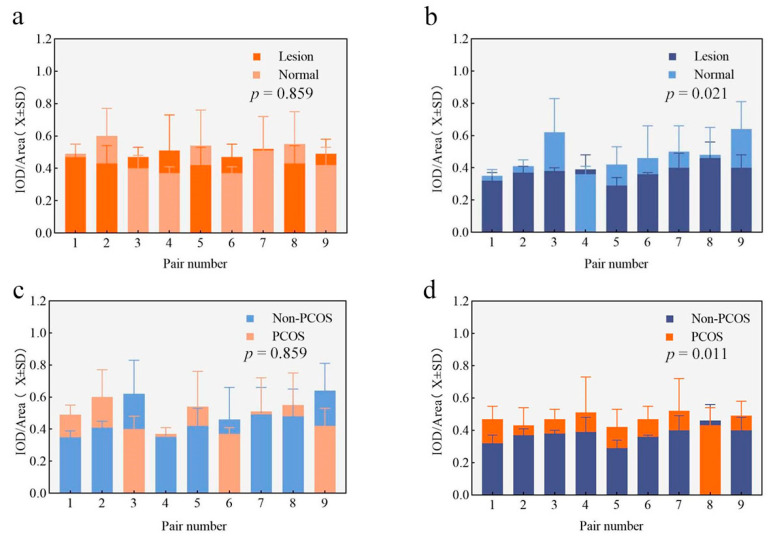
The expression levels of AMHR2 in the endometrium. (**a**) AEH in PCOS women compared to paired controls, (**b**) AEH in non-PCOS women compared to paired controls, (**c**) normal endometrium paired with both PCOS and non-PCOS subjects, (**d**) AEH paired with both PCOS and non-PCOS subjects.

Figure 2 presents an example of AMHR2 immunohistochemical staining in AEH from women with PCOS, alongside their matched normal endometrial control group (scale: 50 μm). Figure 3a illustrates the semi-quantitative results of the immunohistochemical staining and the outcomes of the pair-to-pair comparative analysis.

As depicted in Figure 2, the endometria of AEH in women with PCOS and the matched control group both exhibited AMHR2 protein expression, predominantly localized in the glandular epithelial cells. Furthermore, Figure 3a indicates that there was no statistically significant difference in the expression levels of the AMHR2 protein between the endometrium of AEH and that of the matched control group.

### 3.3. Changes in AMHR2 Expression in AEH Endometrial Specimens of Non-PCOS

Figure 2 presents examples of AMHR2 immunohistochemical staining in AEH among non-PCOS women, alongside their matched normal endometrial control group (scale: 50 μm). Figure 3b illustrates the semi-quantitative results of the immunohistochemical staining and the outcomes of the pairwise comparative analysis. As depicted in Figure 2, both the endometrium of AEH in non-PCOS women and the matched control group exhibited AMHR2 protein expression, predominantly localized in glandular epithelial cells. Furthermore, as indicated in Figure 3b, the expression level of the AMHR2 protein in the AEH endometrium was significantly lower compared to that of the matched control group, with a statistically significant difference noted (*p* = 0.021).

### 3.4. AMHR2 in Endometrium of PCOS and Non-PCOS and Its Comparison

A total of eighteen subjects with PCOS were included in the study, comprising nine subjects with AEH and nine subjects with a normal endometrium. Similarly, eighteen non-PCOS subjects were included, consisting of nine AEH subjects and nine subjects with a normal endometrium. The details of major confounders related to serum AMH levels in both PCOS and non-PCOS subjects, along with the comparative results between groups, are presented in Table 3.

As indicated in Table 3, there were no statistically significant differences in the eight major confounders associated with serum AMH levels (age, BMI, pregnancy history, serum CEA levels, menstrual regularity, dysmenorrhea history, hypertension, and diabetes) between PCOS and non-PCOS subjects, regardless of whether the endometrium was normal or exhibited AEH lesions.

Figure 2 illustrates an example of the AMHR2 IHC staining of the endometrium (both normal and AEH) in PCOS subjects alongside their paired non-PCOS counterparts (scale: 50 μm). As shown in Figure 2c,d, the semi-quantitative results of immunohistochemical staining for the endometrial AMHR2 protein, along with the paired comparative analysis results, were obtained between PCOS and non-PCOS subjects when the endometrium was either in a normal state or exhibited AEH.

In Figure 2c,d, it is evident that the AMHR2 protein was expressed in the endometrium of both AEH lesions in PCOS subjects and their corresponding control group, with localization primarily in glandular epithelial cells. According to Figure 3c, there were no statistically significant differences in AMHR2 protein expression levels in normal endometrial tissues between the PCOS group and the paired non-PCOS group (*p* > 0.05). However, as illustrated in Figure 3d, the expression level of the AMHR2 protein in the endometrium of AEH lesions in PCOS subjects was significantly higher than that in non-PCOS subjects, with a statistically significant difference (*p* = 0.011).

## 4. Discussion

### 4.1. The Expression of AMHR2 in the Endometrium of Reproductive Women and Its Role in the Pathogenesis of EPLs

AMHR2 is expressed in the normal endometrium of healthy women [13,30,31,32], in the endometrium of women with PCOS [31], and during pregnancy [32]. Additionally, AMHR2 expression is observed in the endometrium associated with uterine fibroids [33], adenomyosis [33], endometriosis [12,34,35], EC [8,12,13,14,36], and other uterine diseases.

The expression of AMHR2 in endometrial tissues correlates with cell atypia. In a normal endometrium, the AMHR2 expression is relatively low, whereas it is significantly higher in EH and EC [8,12,13]. Notably, there is no significant difference in AMHR2 expression levels across different EPL categories and the various clinical stages of EC [14]. A study investigating patients with repeated embryo implantation failure found that apoptosis indicators were positively correlated with AMHR2 expression in the endometrium; specifically, the rate of apoptosis in samples with a high AMHR2 expression was significantly greater than in samples with a normal AMHR2 expression [32]. In vitro experiments have demonstrated that AMH can significantly inhibit cell proliferation and promote apoptosis in EC cells [8,14,35,36,37]. Furthermore, prior animal studies have indicated that AMHR2 gene knockout in mice can induce EPLs [38]. These findings collectively underscore the critical role of AMHR2 in the AMH-AMHR cascade signaling pathway and its associated biological effects on tumor inhibition [14].

The results of this study support the inference that significantly reduced serum AMH levels and/or endometrial AMHR2 expression levels attenuate the biological effects of the endometrial AMH-AMHR cascade signaling pathway, thereby contributing to the development of AEH. However, in the investigation of AMHR2 expression levels in the endometrium of reproductive-aged PCOS subjects, no significant difference was observed in the expression level of the AMHR2 protein between the AEH endometrium and the normal endometrial tissue of matched control subjects. These findings necessitate the consideration of other pathogenic mechanisms that may exist within the pathological context of PCOS. It is also necessary to consider the hypothesis that the relatively low biological efficacy of the AMHR2-mediated AMH-AMHR cascade signaling pathway in the endometrium of patients with PCOS results in the loss of AMH’s effect of inhibiting abnormal endometrial proliferation in AEH. Additionally, it was noted that the expression level of the AMHR2 protein in the AEH endometrium of PCOS subjects, characterized by relatively high serum AMH levels, was significantly higher than that in non-PCOS subjects with relatively low serum AMH levels. This finding further supports the inference that PCOS patients exhibit elevated serum AMH levels and the reduced biological potency of AMHR2 in the endometrium, leading to a weakened AMH-AMHR cascade signaling pathway and a consequent loss of the inhibitory effect on AEH.

In vitro experimental studies have investigated the mechanism of the “negative regulation” of the biological function of the target cells’ AMHR2 in environments with elevated AMH levels [39]. The findings indicate that the continuous stimulation of the culture environment with high concentrations of AMH, akin to the elevated serum AMH levels observed in women with PCOS, leads to a compensatory increase in AMHR2 expression within the target cells. However, it is important to note that an increase in AMHR2 protein expression on the effector membrane does not necessarily correlate with an enhancement in the biological functionality of AMHR2. Factors contributing to the abnormal functionality of the AMHR2 protein include structural abnormalities, such as the absence of extracellular domains that specifically bind to AMH, and distribution abnormalities, characterized by clustered distribution with restricted lateral movement. The elevation of these abnormal AMHR2 protein expressions significantly impacts the biological effects of the AMH-AMHR cascade signaling pathway, resulting in a reduced efficiency of AMHR2 expressed by target cells in a high-AMH environment [39]. Furthermore, the phenomenon of “negative regulation” affecting the biological efficacy of the AMH-AMHR cascade signaling pathway does not discount the potential existence of additional promoting mechanisms related to the occurrence of AEH within this signaling pathway. Additionally, the possibility of a synergistic relationship between this signaling pathway and other pathways associated with EPL occurrence cannot be excluded [40].

The findings of this study indicate that the expression level of the AMHR2 protein in the normal endometrium of PCOS subjects is not significantly higher than that in the normal endometrium of matched non-PCOS subjects. This result aligns with previous research on the endometrium of PCOS patients compared to non-PCOS women [31]. In this study, despite considering the underlying pathological status of PCOS subjects, the relatively elevated serum AMH levels and the actual biological activity of AMHR2 in the endometrium did not significantly impact the endometrial AMH-AMHR cascade signaling pathway or its biological effects, allowing the endometrium to maintain a normal state.

### 4.2. The Expression of AMHR2 in the Endometrium Dominated by High Levels of Serum AMH in Reproductive Women with PCOS and EPLs

Notably, serum AMH levels in women with PCOS are reported to be two to four times higher than those in healthy (non-PCOS) women [15,16,41,42,43]. Such elevated serum AMH levels are expected to exert biological effects, such as inhibiting cell proliferation or promoting apoptosis within the endometrial AMH-AMHR cascade signaling pathway.

As a proliferative lesion occurring in tissue derived from Muller’s tube, the endometrium, the risk of an EPL is significantly increased in women with PCOS, being approximately two to six times higher than that of non-PCOS women [17,18,19,44,45,46,47]. This finding appears to contradict the more efficient AMH-AMHR cascade signaling pathway that is associated with elevated serum AMH levels in the endometrium of PCOS patients, which typically promotes significant biological effects such as the inhibition of target cell proliferation and the promotion of apoptosis. For individuals with PCOS, it has been suggested that “the endometrial AMH-AMHR cascade signaling pathway is incomplete or exhibits a functional defect at a certain link [48]”, which may account for this apparent contradiction.

Studies utilizing mouse models with AMHR2 gene editing have demonstrated that the functional knockout of AMHR2 can trigger EPLs [38]. Furthermore, research has indicated that women with PCOS often possess multiple variants of the AMH and/or AMHR2 genes, which negatively impact the AMH-AMHR cascade signaling pathway and its functionality [49]. Additionally, other studies have reported that the expression of low-bioactive AMHR2 in target cells is stimulated under conditions of high AMH levels [39]. The physical environment associated with PCOS, characterized by elevated serum AMH levels and other factors, can directly or indirectly influence the endometrial AMH-AMHR cascade signaling pathway and its biological functions, with the interplay of these multiple factors potentially contributing to an increased risk of EPLs.

The present study found that serum AMH levels in AEH were significantly reduced in subjects with PCOS compared to matched normal endometrial subjects, while the corresponding expression levels of the AMHR2 protein were not statistically significant. These results suggest a compensatory increase in AMHR2 protein expression in AEH among women with PCOS when compared to the paired normal endometrium. However, the biological efficacy of the AMH-AMHR signaling cascade, which includes the inhibition of cell proliferation and the promotion of apoptosis, was relatively low and was also associated with an increased risk of AEH. Additionally, this study demonstrated that the expression level of the AMHR2 protein in the AEH endometrium was significantly higher in PCOS subjects than in matched non-PCOS women. These findings indicate that AMHR2 protein expression in the AEH endometrium of women with PCOS is compensatorily elevated compared to that of matched non-PCOS women. Nevertheless, the low biological efficacy of the AMH-AMHR signaling pathway, including its roles in inhibiting cell proliferation and promoting apoptosis, further exacerbates the risk of AEH.

The elevated serum AMH levels in women with PCOS may initiate their own compensatory protection or prevention mechanisms in the pathogenesis of AEH through the endometrial AMH-AMHR cascade signaling pathway [31,50]. When the physical environment or the physical and mental state of individuals with PCOS exhibits certain characteristics or reaches specific thresholds (such as age, BMI, serum AMH levels, a PCOS phenotype, etc.), or when combined with high-risk factors for EPLs in PCOS (such as infrequent ovulation or anovulation, chronic endometrial inflammatory changes, hyperandrogenemia, abnormal glucose and lipid metabolism, infertility, hypertension, etc.), the endometrial AMH-AMHR cascade signaling pathway and the corresponding expression of AMHR2 (both in terms of the quantity and biological efficacy) may lose their compensatory protection or prevention mechanisms, ultimately resulting in AEH lesions [51,52,53,54,55,56,57,58].

Based on the view that the “serum AMH level is only a relevant factor or auxiliary condition for the occurrence and development of EPL in female individuals, but not a necessary factor or sufficient condition” [51,59], it can be posited that relatively high serum AMH levels may serve as a self-protective or preventive mechanism against the occurrence and development of AEH in women with PCOS. This effect is mediated through the endometrial AMH-AMHR cascade signaling pathway and associated biological effects, such as the inhibition of cell proliferation and the promotion of apoptosis. However, it is important to note that this self-protective mechanism is not an infallible measure or an independent protective factor against the occurrence of AEH. Notably, a higher proportion of women with PCOS can still develop EPLs despite having elevated levels of serum AMH [51,59]. In future studies investigating the pathogenesis of EPLs in women with PCOS, it will be essential to consider the influence of the AMH-mediated endometrial AMH-AMHR cascade signaling pathway and its associated biological effects on the occurrence and development of EPLs. Additionally, attention should be directed towards the disease-causing effects and preventive targets of the various physical and mental environmental risk factors and mechanisms mentioned above. This approach will enhance relevant awareness and provide more accurate and detailed scientific references for the prevention and control of EPLs.

### 4.3. Innovations and Limitations

Considering the evident negative correlation between EPL disease and serum AMH levels, as well as the significantly elevated serum AMH levels observed in women with clinical PCOS compared to their non-PCOS counterparts, this study examined the expression level of AMHR2 in the endometrium of EPL women of childbearing age. The subjects were stratified based on the presence of PCOS, dividing them into PCOS and non-PCOS groups. Subsequently, AMHR2 expression levels in the endometrial specimens of varying pathological types (AEH and normal) from both cohorts were matched, and case–control studies were conducted, accounting for major confounders related to serum AMH levels. The objective of this study was to explore the association between endometrial AMHR2 expression and the development of AEH in distinct environments, given the significantly different peripheral serum AMH levels between PCOS and non-PCOS women. Unlike previous studies [8,12,13,14], this research specifically focused on women of childbearing age. The findings revealed that the expression level of the AMHR2 protein in the AEH endometrium of PCOS women did not differ significantly from that in the normal endometrium of PCOS women but was notably higher than in the AEH endometrium of non-PCOS women. These results are robust and provide valuable insights for the further understanding of the relevant information.

As a preliminary single-center retrospective paired case–control study, this investigation employed the propensity score assessment and allocation pair method for matching. However, it exhibited certain research limitations, including a relatively restricted source of research subjects, a singular detection method, and a limited number of sample pairs. These factors may introduce varying degrees of selection bias and reference bias risks in the resultant findings. The expression level of AMHR2 in the endometrium of AEH cannot be directly inferred from the expression levels of AMHR2 in the endometrium of other EPL categories or the potential dynamic changes among different categories. Future research should involve prospective, multi-center studies with larger sample sizes and multi-platform approaches, encompass all EPL categories, standardize test results, and further verify, supplement, and refine relevant information, so as to mitigate the risk of selection bias to an acceptable level, and, thereby, to possibly obtain better reference values and research and development prospects in the topic presented in this study.

## 5. Conclusions

The AMHR2 protein was expressed in the endometrial tissues of both PCOS and non-PCOS women of reproductive age. In the absence of PCOS, the serum AMH levels and endometrial AMHR2 protein expression in patients with AEH were lower than those in women of reproductive age without endometrial lesions. These findings underscore the significant role of the AMH-AMHR cascade signaling pathway in the endometrium and its associated biological effects in the pathogenesis of AEH. In AEH patients with PCOS, serum AMH levels and AMHR2 protein expression in the lesion endometrium were higher than those in AEH patients without PCOS. This indicates that the presence of PCOS may directly or indirectly influence the AMH-AMHR cascade signaling pathway in the endometrium, thereby affecting the biological effects of this pathway in inhibiting cell proliferation or promoting cell apoptosis. The results and conclusions presented here are only a preliminary exploration of how the AMH-AMHR cascade signaling pathway may impact the occurrence and progression of AEH. Further prospective, multi-center studies with large sample sizes and diverse detection platforms are necessary to verify, supplement, and enhance the relevant information, thereby improving its research and development reference value.

## Figures and Tables

**Table 1:** Main confounding factor levels and comparison list of serum AMH levels in PCOS research subjects.

Characteristics	PCOS-AEH Group		PCOS Control Group		*p* Value *
	Number of Samples	M (P25, P75) or *n* (%)	Number of Samples	M (P25, P75) or *n* (%)	
History of gestation	9	0 (0.0%)	9	0 (0.0%)	>0.05
BMI (kg/m^2^)	9	25.47 ± 3.51	9	23.63 ± 4.64	>0.05
PCOS phenotype	9		9		>0.05
A	3	3 (33.3%)	3	3 (33.3%)	
B	2	2 (22.2%)	2	2 (22.2%)	
C	2	2 (22.2%)	2	2 (22.2%)	
D	2	2 (22.2%)	2	2 (22.2%)	
Hypertension	9	0 (0.0%)	9	0 (0.0%)	>0.05
Diabetes	9	0 (0.0%)	9	0 (0.0%)	>0.05

*: *t* test or chi-square test for paired samples. AMH: anti-Müllerian hormone; PCOS: polycystic ovarian syndrome; BMI: Body Mass Index.

**Table 2:** Main confounding factor levels and comparison list of serum AMH levels in non-PCOS research subjects.

Characteristics	Non-PCOS-AEH Group		Non-PCOS-Control Group		*p* Value *
	Number of Samples	M (P25, P75) or *n* (%)	Number of Samples	M (P25, P75) or *n* (%)	
Age (years old)	9	36.11 ± 4.91	9	32.22 ± 7.00	>0.05
BMI (kg/m^2^)	9	23.65 ± 2.74	9	23.38 ± 3.61	>0.05
CEA (ng/mL)	9	1.26 ± 0.70	9	1.31 ± 0.86	>0.05
Menstrual regularity	9	4 (44.4%)	9	4 (44.4%)	>0.05
History of dysmenorrhea	9	1 (11.1%)	9	1 (11.1%)	>0.05
Hypertension	9	0 (0.0%)	9	0 (0.0%)	>0.05
Diabetes	9	0 (0.0%)	9	0 (0.0%)	>0.05

*: *t* test or chi-square test for paired samples. AMH: anti-Müllerian hormone; PCOS: polycystic ovarian syndrome; BMI: body mass index.

**Table 3:** Main confounders of serum AMH levels in PCOS and non-PCOS subjects and comparison list.

Characteristics	Atypical Endometrial Hyperplasia			Normal Endometrium		
	PCOS (*n* = 9)	Non-PCOS (*n* = 9)	*p* Value *	PCOS (*n* = 9)	Non-PCOS (*n* = 9)	*p* Value *
	M (P25, P75) or *n* (%)	M (P25, P75) or *n* (%)		M (P25, P75) or *n* (%)	M (P25, P75) or *n* (%)	
Age (years old)	35.67 ± 3.87	36.11 ± 4.91	>0.05	30.78 ± 4.32	32.22 ± 7.00	>0.05
BMI (kg/m^2^)	25.47 ± 3.51	23.65 ± 2.74	>0.05	23.63 ± 4.64	23.38 ± 3.61	>0.05
History of gestation	2 (22.2%)	2 (22.2%)	>0.05	2 (22.2%)	2 (22.2%)	>0.05
CEA (ng/mL)	1.50 ± 0.88	1.26 ± 0.70	>0.05	1.38 ± 0.82	1.31 ± 0.86	>0.05
Menstrual regularity	4 (44.4%)	4 (44.4%)	>0.05	4 (44.4%)	4 (44.4%)	>0.05
History of dysmenorrhea	1 (11.1%)	1 (11.1%)	>0.05	1 (11.1%)	1 (11.1%)	>0.05
Hypertension	0 (0.0%)	0 (0.0%)	>0.05	0 (0.0%)	0 (0.0%)	>0.05
Diabetes	0 (0.0%)	0 (0.0%)	>0.05	0 (0.0%)	0 (0.0%)	>0.05

*: *t* test or chi-square test for paired samples. AMH: anti-Müllerian hormone; PCOS: polycystic ovarian syndrome; BMI: body mass index.

## Data Availability

The data sets generated for this study are available on request from the corresponding author.

## Supplementary Materials

The following supporting information can be downloaded at https://www.mdpi.com/article/10.3390/diagnostics14242872/s1: Table S1: Clinical characteristics and comparison of PCOS subjects of reproductive age; Table S2: Clinical characteristics and comparison of non-PCOS subjects of reproductive age.

## Abbreviations

AEH: atypical endometrial hyperplasia; AMH: anti-Mullerian hormone; AMHR: anti-Mullerian hormone receptor; AMHR1: anti-Mullerian hormone receptor type I; AMHR2: anti-Mullerian hormone receptor type II; BMI: body mass index; CEA: carcinoma embryonic antigen; EC: endometrial carcinoma; EH: endometrial hyperplasia; EPLs: endometrial proliferative lesions; FSH: follicular stimulating hormone; HA: hyperandrogenism; IHC: immunohistochemistry; IOD/Area: integral optical density/area; non-AEH: endometrial hyperplasia without atypia; OD: ovulatory dysfunction; PCOM: polycystic ovarian morphology; PCOS: polycystic ovarian syndrome; POI: premature ovarian insufficiency; WHO: World Health Organization.
