# Supplementary material for: PCOS Influences the Expression of AMHRII in the Endometrium of AEH During the Reproductive Age
Source: Diagnostics (Basel). 2024 Dec 20;14(24):2872. doi: 10.3390/diagnostics14242872 (PMC11675281; doi:10.3390/diagnostics14242872)
Supplement: Supplementary file 1 [file diagnostics-14-02872-s001.zip › Supplementary Table S1.pdf]

Supplementary Table S1: Clinical characteristics and comparison of PCOS subjects in reproductive age

Characteristics	PCOS-control group	PCOS-EPL group	*p* value*
	M (P25, P75) or n (%)	M (P25, P75) or n (%)	
Basic characteristics			
Age (years)	30 (28 - 33)	30.5 (29 - 35)	0.067
Age of menarche (years)	13 (13 - 14)	13 (13 - 14)	0.417
Menstrual period (days)	6 (5 - 7)	7 (5 - 7)	0.176
Pregnancy history	688 (61.5 %)	30 (33.3 %)	<0.001
Birth history	252 (22.5 %)	20 (22.2 %)	0.948
BMI (kg/m^2)#	22.27 (20.32 - 24.54)	25.33 (22.17 - 29.10)	<0.001
PCOS phenotype			0.004
A	376 (33.6 %)	23 (25.6 %)	
B	244 (21.8 %)	19 (21.1 %)	
C	112 (10.0 %)	2 (2.2 %)	
D	387 (35.6 %)	46 (51.1 %)	
Basic Diseases			
Hypertension	23 (2.1 %)	15 (16.7 %)	<0.001
Diabetes	24 (2.1 %)	10 (11.1 %)	<0.001
Higher Education	706 (63.1 %)	54 (60.0 %)	0.559
Menstrual Regularity&	374 (33.4 %)	11 (12.2 %)	<0.001
Dysmenorrhea History	267 (23.9 %)	12 (13.3 %)	0.032
Cancer Family History	65 (5.8 %)	7 (7.8 %)	0.448
Smoking History	9 (0.8 %)	0 (0.0 %)	0.828
**Detection Indicators**			
AMH (ng/mL)	7.99 (6.24 - 10.42)	5.01 (2.61 - 7.01)	<0.001
TSH (mIU/L)	1.53 (1.11 - 2.13)	1.61 (1.26 - 2.26)	0.214
PRL (ng/mL)	14.60 (11.00 - 19.80)	14.20 (10.30 - 19.50)	0.368
bLH (IU/L)	7.63 (5.25 - 11.84)	8.17 (5.54 - 11.78)	0.834
bFSH (IU/L)	5.81 (4.99 - 6.79)	6.24 (5.28 - 7.21)	0.129
bE_2 (pmol/L)	106.60 (74.43 - 134.00)	126.50 (103.93 - 150.18)	0.003
bP (nmol/L)	0.95 (0.59 - 1.34)	0.75 (0.41 - 1.13)	0.014
TT (nmol/L)	1.10 (0.80 - 1.50)	1.00 (0.70 - 1.40)	0.177

**Cancer biomarkers**			
CEA (ng/mL)	1.10 (0.70 - 1.50)	1.20 (0.85 - 1.90)	0.060
AFP (ng/mL)	2.20 (1.60 - 3.20)	2.30 (1.50 - 3.55)	0.766
CA125 (U/mL)	14.20 (10.10 - 20.50)	14.95 (11.58 - 22.13)	0.077
CA153 (U/mL)	8.10 (5.90 - 11.70)	8.50 (6.48 - 11.75)	0.318
CA199 (U/mL)	8.90 (5.80 - 13.80)	8.50 (5.10 - 13.10)	0.313

*: Two independent samples Mann-Whitney U test, chi-square test or Fisher's exact test.

#: BMI calculation method: weight divided by height squared, unit is kg/m^2.

&: Menstrual regularity refers to a menstrual cycle of 21 to 35 days, and a menstrual period of 3 to 7 days at the same time.
