# Supplementary material for: PCOS Influences the Expression of AMHRII in the Endometrium of AEH During the Reproductive Age
Source: Diagnostics (Basel). 2024 Dec 20;14(24):2872. doi: 10.3390/diagnostics14242872 (PMC11675281; doi:10.3390/diagnostics14242872)
Supplement: Supplementary file 1 [file diagnostics-14-02872-s001.zip › Supplementary Table S2.pdf]

Supplementary Table S2: Clinical characteristics and comparison of non-PCOS subjects in reproductive age

Characteristics	Non-PCOS-control group<br>M (P25, P75) or n (%)	Non-PCOS-EPL group<br>M (P25, P75) or n (%)	*p* value*
Basic characteristics			
Age (years)	31 (28 - 34)	33 (28 - 38)	0.001
Age of menarche (years)	14 (13 - 14)	13 (13 - 14)	0.087
Menstrual period (days)	6 (5 - 7)	6 (5 - 7)	0.547
Pregnancy history	3394 (64.7 %)	66 (56.4 %)	0.065
Birth history	343 (6.5 %)	6 (5.1 %)	0.542
BMI (kg/m^2)[#]	21.23 (19.53 - 23.03)	23.63 (21.20 - 26.47)	<0.001
Basic Diseases			
Hypertension	37 (0.7 %)	9 (7.7 %)	<0.001
Diabetes	25 (0.5 %)	5 (4.3 %)	<0.001
Higher Education	3262 (62.1 %)	67 (57.3 %)	0.282
Menstrual Regularity[&]	4706 (89.7 %)	76 (65.0 %)	<0.001
Dysmenorrhea History	1496 (28.5 %)	15 (12.8 %)	<0.001
Cancer Family History	297 (5.7 %)	6 (5.1 %)	0.806
Smoking History	52 (0.9 %)	0 (0.0 %)	0.630
**Detection Indicators**			
AMH (ng/mL)	3.28 (2.28 - 4.58)	1.58 (1.11 - 2.36)	<0.001
TSH (mIU/L)	1.52 (1.08 - 2.09)	1.64 (1.13 - 2.37)	0.152
PRL (ng/mL)	15.90 (11.80 - 21.10)	18.00 (12.05 - 20.45)	0.544
bLH (IU/L)	4.70 (3.56 - 5.94)	4.20 (2.59 - 6.17)	0.125
bFSH (IU/L)	6.45 (5.52 - 7.50)	6.38 (4.83 - 8.73)	0.890
bE_2 (pmol/L)	102.80 (75.24 - 132.40)	125.50 (92.00 - 147.60)	0.016
bP (nmol/L)	1.04 (0.70 - 1.41)	0.73 (0.47 - 1.12)	<0.001
TT (nmol/L)	0.70 (0.50 - 1.00)	0.80 (0.50 - 0.90)	0.478

**Cancer biomarkers**			
CEA (ng/mL)	1.10 (0.70 - 1.50)	1.20 (0.80 - 1.70)	0.023
AFP (ng/mL)	2.30 (1.60 - 3.10)	2.50 (1.55 - 3.10)	0.418
CA125 (U/mL)	16.30 (11.60 - 22.10)	17.70 (13.15 - 23.60)	0.055
CA153 (U/mL)	9.20 (6.40 - 11.10)	9.50 (6.70 - 12.25)	0.050
CA199 (U/mL)	10.50 (6.60 - 14.20)	11.90 (7.30 - 14.55)	0.127

*: Two independent samples Mann-Whitney U test, chi-square test or Fisher's exact test.

#: BMI calculation method: weight divided by height squared, unit is kg/m^2.

&: Menstrual regularity refers to a menstrual cycle of 21 to 35 days, and a menstrual period of 3 to 7 days at the same time.
